# Temporal variation in confirmed diagnosis of fever-related malarial cases among children under-5 years by community health workers and in health facilities between years 2013 and 2015 in Siaya County, Kenya

**DOI:** 10.1186/s12936-017-2100-9

**Published:** 2017-11-09

**Authors:** Donald O. Apat, John M. Gachohi, Mohamed Karama, Jusper R. Kiplimo, Sonia E. Sachs

**Affiliations:** 1 Columbia Global Centers | Nairobi, P.O. Box 51412, Nairobi, 00100 Kenya; 2 School of Public Health, Jomo Kenyatta University of Agriculture and Technology, P.O. Box 62000, Nairobi, 00200 Kenya; 3 Umma University, P.O. Box 713, Kajiado, 01100 Kenya; 4 International Livestock Research Institute (ILRI), Old Naivasha Rd, Kabete, P.O. Box 30709, Nairobi, 00100 Kenya; 5 The Earth Institute at Columbia University, 475 Riverside Drive, Suite 1040, New York, NY 10115 USA

**Keywords:** Fever, Malaria diagnosis, Community health worker, Health facilities, Siaya, Kenya

## Abstract

**Background:**

Malaria case management continues to experience dynamic changes. Building community capacity is instrumental in both prevention and treatment of malaria. The World Health Organization (WHO) recommends utilization of well-trained and supervised community health workers (CHWs) to reduce the burden of malaria deaths among children under-5 years of age in Africa. Longitudinally-tracked information on utilization of CHWs by communities in terms of trends in diagnosis of malaria in children under-5 years of age is essential in influencing national and local malaria control policies and strategies.

**Methods:**

A desktop review was carried out of a database consisting of confirmed uncomplicated malaria cases in 10 villages using CHWs and out-patient departments of 10 health facilities in children under-five for the period of 3 years between January 2013 and December 2015. Analyses of association between the diagnosed cases and satellite-based rainfall, village and time (months and years) were carried out using a Poisson regression model.

**Results:**

Analysis of malaria diagnoses made by CHWs showed the following trends: (i) the incidence of reported documented malaria-positive fever cases increased with time (2013–2015) and the difference over the years was statistically significant (*P* < 0.001), (ii) specific village was significantly associated (*P* < 0.001) with reporting malaria-positive fever cases, (iii) the long-term monthly sequence starting from highest to lowest incidence of reported malaria-positive fever cases was July, May and June, March, August, April, September, November, and February, October and, finally, January, and the difference in reported malaria-positives between the months was statistically significant (*P* = 0.001) and (iv) none of the tested rainfall regimes (current, lagged or cumulative) was associated with reported malaria-positive fever cases during the 3-year period (*P* > 0.1). Looking at the number of diagnoses made at the health facilities, (i) The number of reported malaria-positive fever cases decreased with time (2013–2015) and the difference among the years was not statistically significant (*P* = 0.399), (ii) The long-term monthly sequence starting from highest to lowest number of reported malaria-positive fever cases was July, June, May, April, January, August, March, February, September, November, October and December, and the difference between the months was statistically significant (*P* < 0.001).

**Conclusions:**

CHWs have the potential to play a major role in diagnosing and treating malaria, thereby decreasing under-five children mortality. Temporally, the risk of diagnosing malaria seems predictable and this may present opportunities for policy-targeted malaria preparedness and control. The findings are expected to support policy actions that may scale-up community health services in remote rural settings.

## Background

Fever is the most well-recognized sign of an infection and is defined as an elevated axillary temperature (≥ 37.5 °C) in a child or an adult [1]. Fever accounts for over 30–50% of all paediatric cases seeking healthcare in low-resource areas in sub-Saharan Africa [2]. It is also estimated that 28% of fever cases among children aged between 0 and 4 years are likely to seek treatment in a public sector clinic in Africa [3]. In Kenya, fever is the most common symptom exhibited by people seeking health care [4, 5]. Fever is a *sine qua non* of malaria [2], and in turn, malaria is a major contributor to morbidity and mortality in sub-Saharan Africa [6]. In Kenya, malaria is estimated to cause approximately 20% of all deaths of children under 5 years of age [7]. As most health care workers in Africa associate fever with malaria, rapid diagnostic tests (RDTs) for malaria have been developed to improve the rational treatment of children with fever [8]. This management has, over time, realized major changes in recommendations with the aim of reducing the associated morbidity and mortality from malaria [9–11]. One of the recommendations is the integration of community-based health workers (CHWs) into the primary health care systems [12].

Early diagnosis and rapid effective therapy are essential management strategies for surveillance, control and elimination, thus averting malaria morbidity and mortality. Indeed, early effective anti-malarial treatment may also decelerate transmission in a population. According to the World Health Organization (WHO), building community capacity through training and supervising community health workers (CHWs) can help reduce morbidity and mortality associated with malaria in Africa. This can be achieved through rapid and effective treatment of malaria cases within 24 h [12]. In addition, the WHO recommendation on the diagnosis of malaria is based on parasitological diagnostic approaches namely light microscopy and immunochromatographic rapid diagnostic tests (RDTs) [10]. Given the limitations of microscopy in the rural areas, malaria RDT is a user-friendly and accurate diagnostic tool that can be performed at the point of care in communities by CHWs in Africa [13].

Increased utilization of CHWs in the management of malaria and other childhood infections has been comprehensively reported [14–21]. These reports suggest that properly supervised CHWs can lead to improved management of uncomplicated child fever cases in areas with limited health facilities, thereby reducing child mortality. However, literature on characterization of the space–time pattern of utilization and performance of CHWs in terms of diagnosis of malaria carried out in a longitudinal manner is scarce. Lack of longitudinal data on CHWs utilization and performance implies that governments and research partners cannot adequately evaluate the impact of CHWs on health outputs and outcomes. The presence of longitudinal data may also unravel existing and dynamic patterns of acceptability of CHWs by the communities they serve. Furthermore, without data that spans across years, governments and research partners cannot assess the global trends and progress made over time. This study set out to address these concerns from an evidence-based perspective.

Malaria is a complex disease and its transmission and prevalence are influenced by many factors. For instance, rainfall, temperature, and humidity are considered to play a major role in determining mosquito reproduction and mortality [22–24]. Temperature and rainfall influence both the development of mosquitoes and malaria parasites. Increased precipitation generates more breeding grounds for mosquitoes and subsequently increases their numbers [25]. Thus, a pattern exists where periods of low and high risks can be characterized. Where CHWs complement health facilities, data from community-based surveillance can be utilized to describe spatial and temporal patterns of variation in infection or disease incidence [26]. Evidence is, therefore, needed to show whether CHWs can innovatively and effectively play a role in these strategies.

Malaria is the commonest cause of fever in western Kenya where transmission is high with an entomological inoculation rate (EIR) of approximately twenty-four infective bites per person per year with children under-five bearing the brunt [27]. The “Millennium Villages Project” located in this region is an integrated rural development approach initiated in 2004. Professionalized CHWs is one aspect of the “Millennium Villages Project” health system aimed at scaling up of community health delivery.

To address the aforementioned issue of data needs and evidence-based health systems evaluation, this study aimed at determining trends (over a period of 3 years) in the number of fever cases diagnosed as malaria using CHWs in the “Millennium Villages Project” and in health facilities in Yala Division, Siaya County in Kenya. An attempt was also made to determine the periods (months and years) with highest risks of fever which was diagnosed as malaria by the two institutions (CHWs and in health facilities). The study also sought to assess the relationship between fever-related malarial cases and rainfall in the study area. Integrating climatic information enriched this study in determining the role of rainfall patterns in malaria infections over time and/or space and whether these dynamics were implicitly captured in cases diagnosed by the two institutions. The data used was obtained for the period between January 2013 and December 2015 from both 158 CHWs and outpatient departments (OPD) of 10 health facilities in Yala Division, Siaya County in Kenya. CHWs managed uncomplicated malaria cases with artemisinin-based combination therapy (ACT) and referred malaria-negative cases to clinics or hospitals. The study findings will be used to support policy actions towards scaling-up community health services in the management of malaria in resource-limited settings.

## Methods

### Study area

**Fig. 1:**
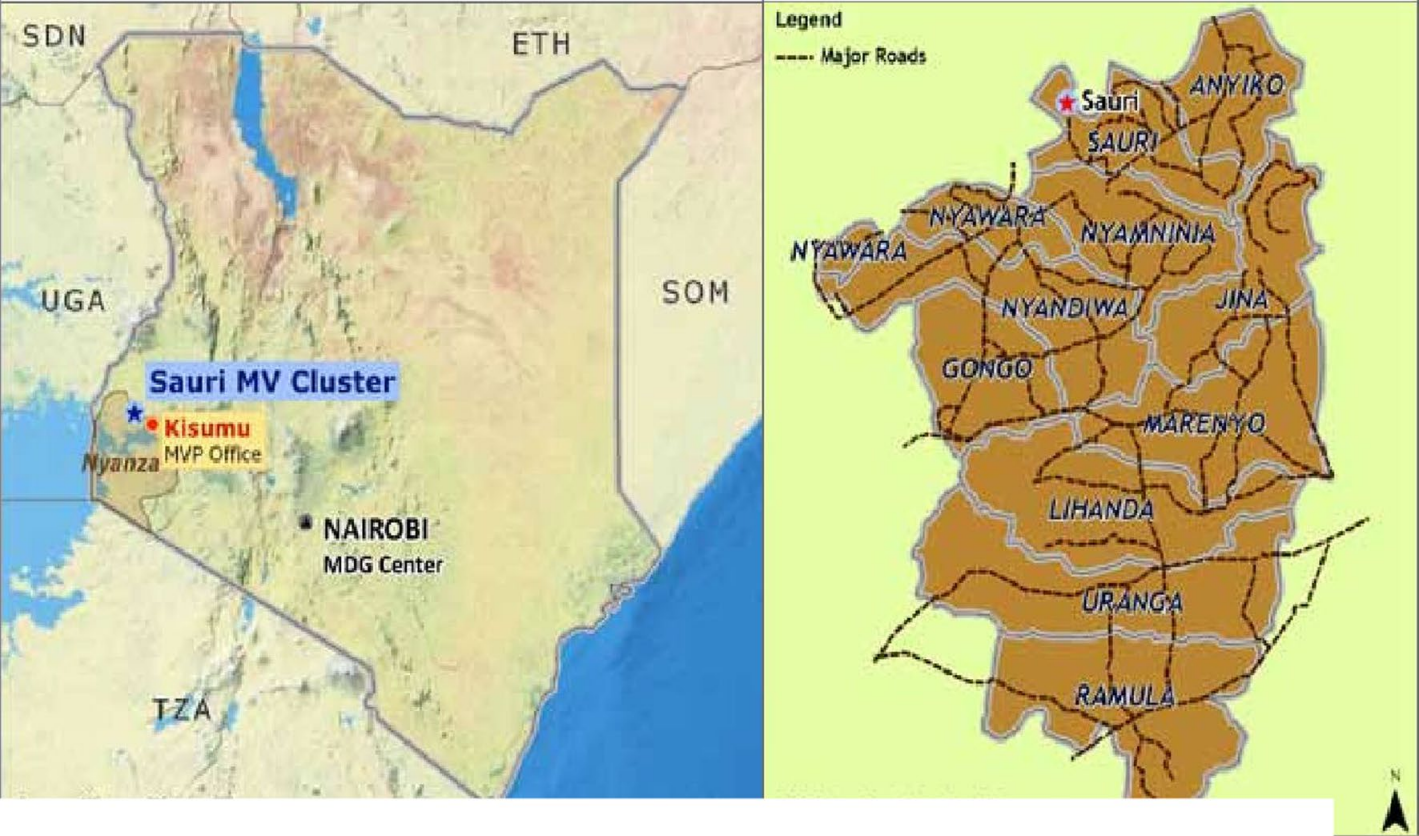
Map of the millennium village cluster and sub-villages, in Siaya County, Kenya

The study was conducted within the “Millennium Villages Project” in the highlands of Western Kenya, in Yala Division, Gem sub-county, in Siaya County. The Millennium Village comprises 11 sub-villages with a total population of approximately 65,000. The site is located at 34.75° longitude east and 0.24° latitude north, 30 km north of Lake Victoria and 1400–1500 m above sea level (Fig. 1). The average temperature in the area is 24 °C, ranging from 18 to 27 °C with an annual rainfall of 1800 mm. The rainfall pattern is bimodal: the long rainy season occurs from March to June and the short rainy season from September to December. Subsistence agriculture is the main livelihood in the area. *Anopheles funestus* and *Anopheles gambiae* sensu stricto (s.s.) are the main anopheline species found in the study area [27].

### Study context

#### Policy context: Kenya National CHW Programme “Community Strategy”

The Kenya’s community health strategy 2006 [28] institutionalized CHWs into Level one of Kenya’s primary health care delivery system by clearly providing constructs that operationalize service provision at the community level. The operationalization includes establishing a Level 1 care unit to serve a local population of 5000 people by instituting a cadre of well-trained CHWs: each worker provides Level 1 service to 20 households; one community health extension worker (CHEW) supports 25 CHWs and ensures that the recruitment and management of CHWs is carried out by village and facility health committees [28]. CHEWs also support CHW through supervision and coaching and meet with their CHWs monthly [29].

Parts of the Community Strategy were revised in 2010 following resolutions of the Ministry of Health, Health Sector Coordinating Committee (HSCC). Household coverage was revised to correspond with the population density ranging from one CHW covering 500 people for areas with dense populations. The policy document also stipulated that the CHWs were entitled to a minimum payment of Kenya Shillings 2000 (US$20) a month as a performance-based incentive [30].

The CHWs conjoin national health facilities and the community members with specific responsibilities such as health promotion, disease prevention, care seeking and treatment of specific diseases, such as uncomplicated malaria and diarrhea and compliance with treatment and advice and household follow-up [28].

#### The Millennium Villages Project approach

The “Millennium Villages Project” (MVP) was a demonstration project of the Earth Institute at Columbia University and a non-governmental organization, Millennium Promise Alliance. The project hypothesized that an integrated approach to rural development could be used to achieve the millennium development goals (MDGs). MDG 8 (Combat HIV/AIDS, malaria and other diseases) recommended targets addressing the challenges by the main burden of disease and to do so by the end of 2015 [16].

The MVP’s CHW programme strategies are described elsewhere [16, 31]. In brief, the CHW programme of the MVP utilizes a workforce of CHWs, with each CHW serving at least 100–150 households and approximately 650 people. The MVP CHWs are supervised by senior CHWs in groups of six. The seniors are in turn supervised by Health Facilitators in a ratio of approximately 8–20 depending on the setting. The CHWs provide preventative care through health education and limited curative services. They are provided with a CHW kit that has basic drugs such as oral rehydration solution, zinc, paracetamol, RDTs for malaria parasites detection in reported fever cases, and artemether-lumefantrine (Coartem^®^) for the household-level treatment of positive RDT cases. Children under-5 years receive monthly routine follow-up for danger signs detection, growth monitoring assessments, use of bed nets for malaria prevention, vitamin A and immunization status. However, for this study, only child presenting with fever was tested for malaria. The CHWs are supported by Information and Communications Technology (ICT) systems that are facilitated through a mobile telephony system. The mobile heath technology uses information collected at the household level by CHWs to monitor child and maternal health, as well as monitor compliance with treatment administered at the clinic level. Currently, within MVP, CHWs are County employed and are considered to be volunteers, although at the time of the study, they were receiving an additional 4000 Kenyan Shillings (~ $40 USD) per month.

### Health facility diagnosis and management

Case management at the government health facilities in the study site was based on WHO and national guidelines for diagnosis and treatment of malaria. Briefly, diagnosis of malaria was carried out with parasitological confirmation using microscopy and/or RDTs. Only confirmed positive cases of uncomplicated malaria were initiated on treatment with ACT. Cases of severe/complicated malaria were given initial treatment but immediately referred by ambulance to a referral hospital for parenteral treatment.

### Database

A desk review of the project database was conducted. Monthly fever entries and confirmed malaria-positive counts presented by CHWs and OPD of the health facilities among children under-5 years of age over a period of 3 years (2013–2015) were extracted. Fever (fewer than 7-days) cases reported were determined based on integration of (1) history from parent/caregiver; or, (2) chills; or, (3) a temperature (axillary) of 37.5 °C or above. Malaria testing with either an RDT kit or microscopic blood examination was administered for all fever cases. A positive RDT or microscopic examination was classified as a malaria case and a complete dose of ACT was given based on age.

### Precipitation data

Malaria transmission is rainfall sensitive and, therefore, attempts were made to take advantage of the available supply of satellite-based rainfall information to supplement the scantily available ground-based precipitation data. Monthly satellite rainfall data for the study area and period were obtained from the Tropical Rainfall Measurement Mission (TRMM) [32]. Satellite-based TRMM precipitation estimate has been used in predicting and simulating mosquito population dynamics and mosquito-borne disease risk [33]. Different regimes of rainfall were assessed for the association with fever and malaria counts—current study month estimate, 1-, 2- and 3-month lagged values and 2- and 3-month cumulative rainfall.

### Descriptive analyses

Data were descriptively analysed as proportions of fever and malaria-confirmed cases out of the total population of children aged less than 5 years in a specific village, in a given month. Results were presented in frequency tables and graphs of proportions or counts.

### Inferential analyses

Malaria data from CHW were presented in form of counts per village, by month and year whereas the malaria data from health facilities were presented in form of counts by month and year alone (without village information). In both cases, the data were assumed to follow Poisson distribution which is used to model counts of disease events in a group of individuals. Several forms may be assumed to follow Poisson distribution: (i) count of cases over a period of time with the amount of person-time at risk having to be taken into account; (ii) count of cases of disease with the size of population at risk being taken into consideration; or (iii) a count of outcome that is measured over a geographical area. In this study, count of cases of confirmed malaria with the size of the population at risk being taken into consideration was used.

The Poisson regression model was described as follows: *E(Y)* = *μ* = *nλ* where: *E(Y*) was the expected number of cases of malaria and *n* was the exposure (which adjusted for the different sizes of the children population at risk by month, year and village). In this study, *n* was transformed to a log scale, i.e. the log of the population at risk which is normally referred to as an offset. *λ* represented a function which defined the malaria occurrence. One of the ways that *λ* could be related to the predictor (independent variables)—in this study, rainfall and month of the year and village—was: $\lambda = e^{{\beta_{0} + \beta_{1} x}}$. Consequently, the Poisson model was of the form $E(Y) = ne^{{\beta_{0} + \beta_{1} x}}$ or $\log E(occurrence) = \log \frac{E(occurrence)}{n} = \beta_{0} + \beta_{1} x$ where ln *E*(*occurrence*) was the log of the expected value of the occurrence of malaria cases being modeled as a linear combination of predictors. This formed the univariable analysis (rainfall, month, year and village separately for data from CHWs and month and year separately for data from health facilities).

The Poisson model assumes that the mean and the variance are equal (conditional upon the predictors in the model)—that is, the mean and the variance of counts are equal following consideration of the effects of the predictors in the model. However, the variance may be greater than the mean in the raw data (i.e. unadjusted estimates) and still meet this requirement. Nevertheless, if the unadjusted variance is greater than twice the unadjusted mean, then overdispersion is highly suspected. Overdispersion is said to occur when the variance is much larger than then the mean. This is common with count data and arises when the data are clustered—persons within a village or with time. Thus, in this study, part of variation between villages or unit time (months or years) was due to the variation between villages or time. Consequently, the model will not fit the data well. This study dealt with this problem by fitting a model which allowed the variance to be larger than the mean by assuming that the variance is a function of the mean as follows: var = (1 + *αμ*)*μ* = *μ* + *αμ* ^2^ where *α* was the overdispersion parameter. This formulation gives rise to a negative binomial model. Note that if *α* = 0, then the variance will equal *μ* and the model is a simple Poisson model. The interpretation of a negative binomial distribution as a Poisson distribution with extra dispersion corresponds to a random effects model where the distribution of Poisson means is subjected to additional variation which has a gamma distribution. As with Poisson distribution, the usual form was similar to the expressions provided above for Poisson model except that *E*(*Y*) had a negative binomial distribution. The negative binomial model was fit using an iterative maximum likelihood estimation procedure. The level of significance was set at *P* ≤ 0.1. The statistical significance of the contribution of an individual contributor to the model was tested using likelihood ratio tests (*P* < 0.05). Overdispersion was evaluated using a likelihood ratio test that compared the usual Poisson model to the negative binomial model by testing whether *α* = 0 (level of significance set at *P* ≤ 0.05).

### Ethical approval

Ethical approval was obtained from the Kenya Medical Research Institute (Non-SSC protocol 030) and Columbia University, USA (Protocol: IRB-AAAF1647 [Y3M00]).

## Results

### Diagnosis of fever-related malaria cases by CHWs

**Fig. 2:**
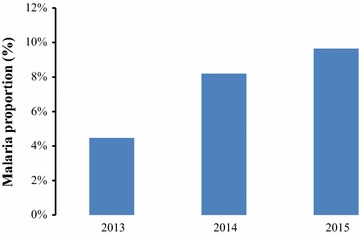
Average proportions of confirmed malaria cases diagnosed by community health workers in children under-5 years

**Fig. 3:**
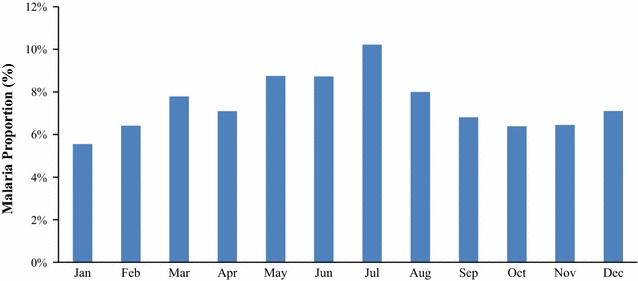
Long-term average monthly proportions of confirmed malaria cases diagnosed by community health workers in children under-5 years

**Fig. 4:**
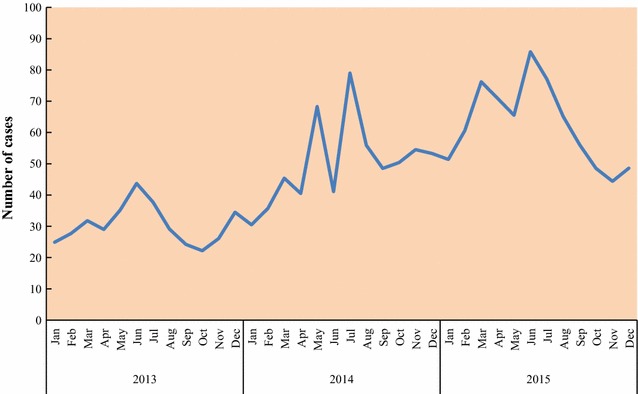
Graph showing monthly counts of confirmed malaria cases diagnosed by community health workers in children under-5 years

Table 1 and 2 shows the (a) average population of children under-5 years of age, (b) mean number fever episodes, (c) mean proportion of children under-5 years of age that developed fever, (d) mean number of confirmed malaria episodes, (e) mean proportion of children under-5 years of age with confirmed malaria, (f) mean number of non-malaria episodes and (g) mean proportion of children under-5 years with non-malaria episodes as reported by community health workers by village and year respectively between years 2013 and 2015 in Siaya County, Kenya. The yearly average population of children under-5 years of age in each of the ten villages ranged between 414 and 936 per village during the study period (Table 1). The mean proportions of fever cases ranged between 5.8 and 13.3% across the villages whereas mean proportions of malaria cases ranged between 4.7 and 12.0% across the villages. Figure 2 shows the proportions of confirmed malaria cases between the years 2013 and 2015. The incidence of malaria-positive cases as diagnosed by CHWs increased with time (Fig. 2). Figures 3 and 4 show the long-term (3-year study period) monthly average proportions of malaria diagnosis by CHWs. The months of July, May, and June had the highest incidence of diagnosed malaria (10.20, 8.74, and 8.73%, respectively in that order). On the other hand, the months of January, October and February had the lowest incidence of diagnosed malaria-positive cases with proportions of 5.55, 6.38, and 6.41% respectively in that order (Figs. 3, 4).

**Table 1:** Descriptive statistics of children under-5 years as reported by community health workers by village

Village	Average population (< 5 years)	Average fever counts	Mean fever proportion (%)	Average malaria counts	Mean malaria proportion (%)	Average non-malaria counts	Mean non-malaria proportion (%)
Anyiko	483.3	39.2	8.3	34.4	7.3	4.8	1.0
Gongo	507.3	51.3	10.4	41.0	8.4	10.3	2.0
Jina	414.0	38.0	9.6	29.9	7.4	8.1	2.2
Lihanda	838.3	63.9	7.7	54.7	6.6	9.2	1.1
Marenyo	925.7	76.4	8.5	69.4	7.7	7.0	0.8
Nyamninia	936.3	56.8	6.1	43.8	4.7	12.9	1.4
Nyandiwa	607.7	34.3	5.8	29.6	5.0	4.7	0.8
Nyawara	406.7	36.4	9.3	31.2	8.0	5.2	1.3
Ramula	764.7	101.7	13.3	92.1	12.0	9.6	1.3
Uranga	719.7	62.6	8.8	51.8	7.3	10.7	1.5

**Table 2:** Descriptive statistics of malaria diagnosis by community health workers by years

Year	Average population (< 5 years)	Average fever counts	Mean fever proportions (%)	Average malaria positive counts	Mean malaria positive proportions (%)	Average non-malaria counts	Mean non-malaria proportions (%)
2013	688.6	39.2	5.8	30.5	4.5	8.6	1.4
2014	631.2	57.8	9.5	50.3	8.2	7.6	1.3
2015	661.3	71.2	11.0	62.5	9.6	8.6	1.3

### Diagnosis of fever-related malarial cases in health facilities

**Fig. 5:**
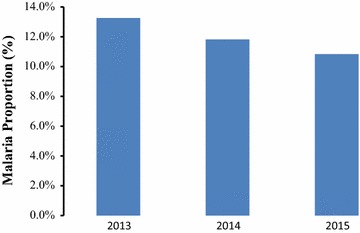
Average proportions of confirmed malaria cases diagnosed in health facilities in children under-5 years

**Fig. 6:**
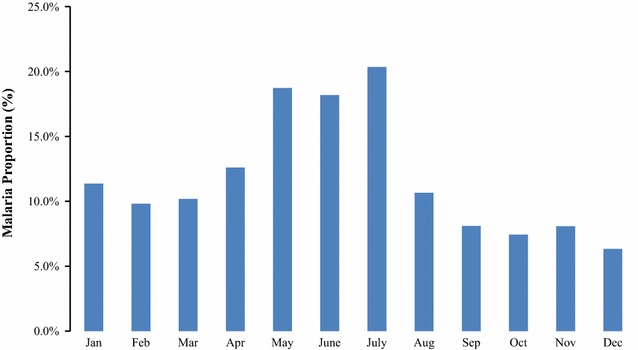
Long-term average monthly proportions of confirmed malaria cases diagnosed in health facilities in children under-5 years

**Fig. 7:**
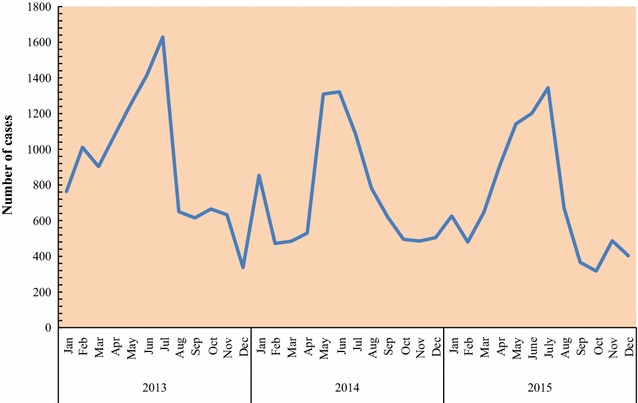
Line graph showing monthly counts of confirmed malaria cases diagnosed in health facilities in children under-5 years

Table 3 shows the (a) average population of children under-5 years of age, (b) mean number fever episodes, (c) mean proportion of children under-5 years of age that developed fever, (d) mean number of confirmed malaria episodes (e) mean proportion of children under-5 years of age with confirmed malaria, (f) mean number of non-malaria episodes and (g) mean proportion of non-malaria episodes in children under-5 years as reported by health facilities between years 2013 and 2015 in Siaya County, Kenya. The mean proportions of fever cases ranged between 22.1 and 25.5% across time whereas mean proportions of malaria cases ranged between 10.8 and 13.3% across time. Figure 5 shows the proportions of confirmed malaria cases between the years 2013 and 2015. The incidence of reported malaria-positive cases in health facilities decreased with time (Fig. 5). Figures 6 and 7 show the long-term monthly average proportions of malaria diagnosis in health facilities. The months of July, May, and June had the highest incidence of reported malaria-positive cases (20.3, 18.7, and 18.2%, respectively in that order). On the other hand, the months of December, October and November had the lowest incidence of reported malaria-positive cases with proportions of 6.3, 7.4, and 8.1%, respectively in that order (Figs. 6, 7).

**Table 3:** Descriptive statistics of children under-5 years as reported in health facilities

Year	Population (< 5 years)	Fever counts	Mean fever proportions (%)	Average malaria positive counts	Mean malaria positive proportions (%)	Average non-malaria counts	Mean non-malaria proportions (%)
2013	6886	1753.3	25.5	912.4	13.3	840.8	12.2
2014	6312	1563.5	24.8	745.7	11.8	817.8	13.0
2015	6613	1462.4	22.1	716.2	10.8	746.3	11.3

### Negative binomial regression univariable analyses for diagnosis of malaria cases by CHWs

Table 4 shows the estimated negative binomial regression coefficient comparing (1) all villages to Anyiko village, (2) years 2014 and 2015 to 2013, and (3) all months of the year to January. Various regimes for the rainfall were evaluated for their relationship and fever-related confirmed malaria-positive cases. Five villages, Ramula, Marenyo, Uranga, Gongo and Lihanda in that order had a significantly higher incidence of reported malaria-positive fever cases relative to Anyiko village (*P* < 0.05) (Table 4). In addition, the village variable was significantly associated (likelihood ratio test *P* < 0.001) with reporting malaria-positive fever cases (Table 4). The incidence of reported malaria-positive fever cases increased with time and the difference among the years was statistically significant (Table 4). Long-term, all months had a higher incidence of reported malaria-positive fever cases relative to January (*P* < 0.05). The higher incidence of reported malaria-positive cases was statistically different from January in the months of March and in the period May to August (*P* < 0.05). The long-term monthly sequence starting with highest to lowest incidence of reported malaria-positive fever cases were July, May and June, March, August, April, September, November, and February, October and finally January. None of the tested rainfall regimes were associated with reported malaria-positive fever cases during the 3-year period. The likelihood ratio tests of *α* for all predictors were all highly significant (*P* < 0.001) implying that the variance in the data was higher than would be expected for a Poisson regression model.

**Table 4:** Negative binomial regression univariable analyses (*P* ≤ 0.1) for cases diagnosed by community health workers

Variable	Variable category	Coefficient	95% confidence interval	P > |z|	Likelihood ratio test P*	*α*	Likelihood-ratio test of α = 0
Village	Marenyo	0.52	[0.30, 0.74]	0.000	0.000	0.21	0.000^╩^
	Nyawara	0.06	[− 0.17, 0.28]	0.608			
	Nyandiwa	− 0.21	[− 0.44, 0.01]	0.064			
	Gongo	0.26	[0.04, 0.49]	0.021			
	Ramula	0.83	[0.61, 1.06]	0.000			
	Nyamninia	0.01	[− 0.21, 0.24]	0.896			
	Jina	− 0.13	[− 0.36, 0.08]	0.234			
	Uranga	0.32	[0.10, 0.54]	0.005			
	Lihanda	0.23	[0.01, 0.46]	0.040			
Year	2014	0.52	[0.39, 0.64]	0.000	0.000	0.22	0.000
	2015	0.73	[0.61, 0.86]	0.000			
Month	February	0.15	[− 0.13, 0.42]	0.301	0.001	0.28	0.000
	March	0.35	[0.07, 0.63]	0.012			
	April	0.26	[− 0.01, 0.54]	0.063			
	May	0.45	[0.18, 0.73]	0.001			
	June	0.45	[0.18, 0.73]	0.001			
	July	0.59	[0.31, 0.87]	0.000			
	August	0.34	[0.06, 0.62]	0.015			
	September	0.18	[− 0.09, 0.46]	0.193			
	October	0.12	[− 0.15, 0.40]	0.374			
	November	0.15	[− 0.12, 0.43]	0.284			
	December	0.24	[− 0.03, 0.52]	0.088			
Rainfall	Current	0.00042	[− 0.0006, 0.0015]	0.436	0.436	0.30	0.000
	1 month lag	− 0.0004	[− 0.0006, 0.0015]	0.410	0.411	0.30	0.000
	2 month lag	0.00014	[− 0.002, 0.002]	0.896	0.896		
	2 month cumulative	− 0.00001	[− 0.0006, 0.0006]	0.964	0.964	0.30	0.000
	3 month cumulative	0.00006	[− 0.0004, 0.0006]	0.825	0.825	0.30	0.000
	4 month cumulative	− 0.00005	[− 0.0005, 0.0004]	0.847	0.847	0.30	0.000

* *P* used to test the statistical significance (*P* ≤ 0.1) of the contribution of the variable to the univariable model; ^╩^ *P* used to test the statistical significance of α (the overdispersion parameter). When the statistical significance of α is significant (*P* ≤ 0.05), it suggests that the variance in the data is higher than would be expected for a Poisson regression

### Negative binomial regression univariable analyses for diagnosis of malaria cases in health facilities

Table 5 shows the estimated negative binomial regression coefficient comparing (1) years 2014 and 2015 to 2013, and (2) all months of the year to January. In contrast with diagnoses by CHWs, the incidence of reported malaria-positive fever cases diagnosed in facilities decreased with time though the difference among the years was not statistically significant (Table 5). In contrast with diagnoses by CHWs, only the months of April, May, June, and July had a higher incidence of reported malaria-positive fever cases relative to January (*P* < 0.05). The higher incidence was statistically different from January for these months (*P* < 0.05) with for the exception of April. Again in contrast with diagnoses by CHWs, 7 months (February, March, August, September, October, November and December) had a lower incidence of reported malaria-positive fever cases relative to January. The lower incidence was statistically different from January for the period between September and December but not in February and March and August (*P* < 0.05). The long-term monthly sequence starting with the highest incidence of reported malaria-positive fever cases was July, June, May, April, January, August, March, February, September, November, October and December. The likelihood ratio tests of *α* for all predictors were all highly significant (*P* < 0.001) implying that the variance in the data was higher than would be expected for a Poisson regression model.

**Table 5:** Negative binomial regression univariable analyses (*P* ≤ 0.1) for cases diagnosed in health facilities

Variable	Variable category	Coefficient	95% confidence interval	P > |z|	Likelihood ratio test P*	*α*	Likelihood-ratio test of α = 0
Year	2014	− 0.16	[− 0.49, 0.17]	0.337	0.399	0.17	0.000^╩^
	2015	− 0.22	[− 0.55, 0.11]	0.188			
Month	February	− 0.13	[− 0.46, 0.18]	0.403	0.000	0.04	0.000
	March	− 0.10	[− 0.42, 0.22]	0.536			
	April	0.11	[− 0.21, 0.43]	0.505			
	May	0.50	[0.17, 0.82]	0.003			
	June	0.56	[0.23, 0.88]	0.001			
	July	0.59	[0.26, 0.91]	0.000			
	August	− 0.06	[− 0.39, 0.26]	0.698			
	September	− 0.33	[− 0.66, − 0.01]	0.044			
	October	− 0.42	[− 0.75, − 0.09]	0.012			
	November	− 0.33	[− 0.66, − 0.01]	0.043			
	December	− 0.58	[− 0.91, − 0.25]	0.000			

* *P* used to test the statistical significance (*P* ≤ 0.1) of the contribution of the variable to the univariable model; ^╩^ *P* used to test the statistical significance of α (the overdispersion parameter). When the statistical significance of α is significant (*P* ≤ 0.05), it suggests that the variance in the data is higher than would be expected for a Poisson regression

## Discussion

This study has demonstrated clear temporal differences in the diagnosis of malaria by CHWs and by the clinicians in health facilities among children under-5 years of age between January 2013 and December 2015 in Siaya County, Kenya. Whereas the diagnoses of malaria cases using household-administered rapid diagnostic test (RDTs) by CHWs increased with time, the diagnoses of malaria in health facilities decreased with time during the study period. This concurs with reported increase in effective utilization of CHWs as a source of advice and/or case management for child fevers at the household level, corresponding with a decline in visits at government facilities and other sources including shops in coastal Kenya [15]. It reflects a gradual shift in the use of health facilities towards the use of CHWs for health care by the community. It also demonstrates emerging health care delivery system where most of the simple malaria cases are detected and treated at the household level. Moreover, the study findings support the proposition that CHWs can play a key role in complementing facility-based health service delivery to communities in developing countries such as Kenya [15]. It is possible that CHWs’ deep and extensive understanding of the community’s context and values and their own permanent residency within the community generates widespread acceptable client-service provider relationships relative to health facility staff [17, 34]. Potential benefits of this shift can be a decrease in the health facility burden by uncomplicated paediatric fever cases and therefore avail health facility staff [17] for other cases particularly non-malarial fever cases. This shift to diagnosis and treatment by CHW increases the proportion of children who receive appropriate treatment for febrile illness and receive it early, within 24 h of onset of symptoms [35]. Emerging professionalized CHW programmes have deliberately and progressively been supported in extending primary health care from facilities to communities in rural and other low-income settings in diverse countries [36]. By integrating CHW programmes into the formal health care delivery systems with training, supervision, reporting and feedback mechanisms, communities may be starting to be sensitive to these programme mechanisms. Given the broader range of services that CHWs provide, by taking advantage of the new technologies in health, qualitative research is needed to understand the experiences and attitudes of communities towards CHW programmes. This would provide evidence to further support scaling up and deeper integrating a formal CHW cadre into health systems of the primary health care delivery. Areas of health that would greatly benefit include surveillance to pick up epidemics early, rapid diagnosis and case management and implementation of preventive measures to diseases which contribute to mortality in sub-Saharan Africa.

Nevertheless, these findings were realized in the context of a project that emphasized, paid, supervised, smartphone empowered, professional cadre of CHWs for improved quality of services, retention, and accountability [16]. Whereas the approach and features of the project could have positively influenced results presented in this paper, the findings are a pointer to the fact that if well supported and motivated, CHW activities can have a profound effect on improving public health in the communities. The cost for scaling up a CHW subsystem was computed to be US$6.56 per head per year for the rural population including the smartphone utilization [37]. This is a demonstration of how the high impact system can be implemented at a low cost that national governments in Sub-Saharan Africa can afford.

Community health workers play an important role in malaria surveillance as part of the broader healthcare system [38, 39]. The primary objective of surveillance is to assess trends over time and identify geographic differences in malaria incidence in real-time so that findings can be quickly addressed and corrected. It is estimated that malaria surveillance systems detect 19% of cases that occur globally [40]. This is because the majority of malaria surveillance reports are derived from health facilities despite a relatively low proportion of patients with fever cases who attend public health facilities. With this inherent limitation in the reporting system in the public sector, low-cost innovative approaches including community-based services [8, 26, 41] are needed in collecting malaria surveillance data which allows for the analysis of spatial distribution of patterns of malaria cases over time. This could help direct intervention efforts and identify areas by village site that are more affected by malaria, identify trends in cases by time and location that require additional interventions and assess the impact of control measures.

In order to further understand why there was an increase in cases detected by CHWs over the study period, possible reasons are described in this paper: (1) decreased availability or use of vector control programmes and dilapidation of the healthcare infrastructure, and (2) climate variability. Malaria is a climate-sensitive disease with temperature influencing the development rates and longevity of malaria parasites and mosquito vectors [23]. In addition, rainfall influences the availability of mosquito aquatic stage breeding sites and, thus, mosquito population dynamics [23]. However, there is no evidence to support substantial climate variability as measured by the average annual variance in the temperature and/or rainfall during the study period to result in the observed increases in detection. In addition, the period of study was too short for detailed analyses on effects of climate variability. On the temporal progressive preference of CHWs, a previous study in Kenya reported diverse barriers on both the demand and delivery of prompt and effective treatment for malaria in children under-5 years of age at government health facilities [42]. Notably, the effects of extreme rainfall on local infrastructure, for instance, flood damage to roads and bridges have a potential to affect community access and mobility to health facilities and shift demand for health care to the closely available CHWs. The authors have no evidence that these factors may have played a role in this context. Moreover, this study period coincided with the period of commencement of the devolved health care service delivery in Kenya and, therefore, dilapidation of the healthcare infrastructure can theoretically be ruled out due to initial enthusiasm about the new health delivery system. In a separate study, an examination of paediatric hospitalizations due to malaria was carried out between 2 and 36 month periods: September 2003 and August 2006 and the period September 2006 to August 2009 [43]. These two periods represented major shifts in intervention policy change and scaled intervention (pre- and post- respectively). Whereas all sites showed a significant reduction in malaria cases between these two time periods, the situation was different in Siaya County where malaria admission rates rose in the second period compared to the pre-scaled intervention period before September 2006 [43]. This suggests that the malaria transmission risk is persistently high in Siaya County and requires attention.

Diagnoses of fever-related malaria by CHWs and in health facilities mostly occurred around July, May, and June in that order across the 3-year study periods. Rainfall in the study area is bimodal—the long rainy season occurs from March to June and the short rainy season from September to December. Studies have reported an association between rainfall and malaria incidence 3–4 months after commencement of rains [44–48]. For instance, in Nandi Highlands in western Kenya, epidemics occurred in 1931, 1932, 1934, 1937, 1940 and 1944, and in each of the years, 1945–1948 malaria was prevalent from May to July [49]. In May 2002, exceptional rainfall in the western highlands of Kenya led to epidemics in some districts in June and July [50] and the months with peak parasite densities appeared to be 1–2 months following rainfall peaks [51, 52]. Predictably, increased rainfall generates multiple breeding sites for mosquitoes, thus increasing their numbers and in turn influencing the vectorial capacity. With this seasonal predictability of malaria transmission, these findings present an opportunity for temporally targeted refresher CHW training, improved capacity in terms of availing prevention strategies, diagnostic kits, and medications.

Interestingly, significant village-scale spatial variations in malaria diagnosis were observed across villages with regard to community-based diagnosis in this study. This could be due to two reasons and/or an interaction between them. The variations may have occurred due to variations in locations of the villages across the study site as also observed by Chanda et al. [53] in Zambia. Spatial epidemiology describes how the temporal dynamics of a host, vector(s), and pathogen populations interact spatially within a permissive environment to enable transmission [54]. In the context of spatial epidemiology in this study, villages on the outer edges of the study area (Ramula, Gongo, and Nyawara) had high cases of malaria compared to villages located in the center of the study area (Nyamninia, Nyandiwa, and Lihanda). Inter-village heterogeneity in local hydrology can generate dissimilar micro-habitats of suitable breeding areas following rain perhaps exhibiting different levels of transmission. Village-scale spatial variability in hydrology may as well be associated with either presence/absence of vegetation which provides resting sites for adult mosquitoes or with topographic effects. In this manner, village-scale hydrological conditions may become important determinants of local malaria transmission [55]. This could further be influenced by the observation that the characteristic spatial scale of Anopheles mosquito population movement is approximately 1–2 km [56], which is approximately equivalent to sizes of villages in Kenya. Indeed, studies have reported that breeding habitats with high transmission intensity frequently occurs within tens to hundreds of meters of the nearest human habitation [57]. Secondly, socio-economic and socio-cultural factors influencing health-seeking behaviour could generate village-level spatial effects. One possible explanation for this is the distance to the CHW versus distance to health centers. Families who are far from health centers are more likely to utilize CHWs than ones close to health centers in the management of uncomplicated malaria [58]. In addition, individual factors in terms of performance of CHWs and corresponding perception of effectiveness may have varied from one village to another. It is also possible that the level of support supervision by support managers was not objectively constant across the villages. This latter reason is supported by studies that evaluated CHW performance in 1998, 1999, and 2001 in this study site—Siaya, Kenya which reported that key reasons for the inadequacies in performance appeared to be guideline ambiguities and weak and subjective clinical supervision [59]. In Ethiopia, socio-economic, geographic and demographic factors were closely associated with the risk of malaria in different villages [60]. This study, therefore, hypothesizes that socio-economic and socio-cultural factors in with community-malaria case management associated with utilization of CHWs may have implications in perceived spatial variations in malaria transmission risk and detection.

In this study, none of the tested rainfall regimes (current, lagged or cumulative) was associated with reporting malaria-positive fever cases during the 3-year period. A similar observation has been reported where other environmental parameters, i.e. vegetation index and surface temperature demonstrated strong associations [33]. Although the association between rainfall and malaria is well established, malaria transmission and the environmental parameters are related in a complex way, e.g. while elevated rainfall may intensify vector populations generating numerous Anopheles breeding sites, excessive rains falling in short time may wash away larvae [61]. In western Kenya, (and Africa in general) weather stations are sparse and data that emanates from is not reliable. This lack of meteorological information was compensated for by using satellite-based methods to source the rainfall data. Previous reports have favourably validated satellite products derived from TRMM sensor with the ground-based weather stations [62]. Moreover, the TRMM has been demonstrated to present a superior spatial and temporal estimate of precipitation in Africa [62] relative to other satellite-based rainfall estimate products. Other reasons that could have influenced the observed lack of relationship in this study include topography, vegetation, soil type differences as well as shallow groundwater behaviour which have been reported to influence malaria transmission at micro-habitat levels [63].

This study is not without limitations. The study did not acquire data that would increase the understanding of factors associated with utilization of CHWs relative to utilization of health facilities. This would include data that would describe characteristics of families [15], utilization of shopkeepers in the study area [15], perceived strengths and weaknesses of CHWs and knowledge, attitude and practices of malaria diagnosis and treatment. A key question is whether increased utilization of CHWs and increased diagnosis of malaria is associated with improved access to prompt and effective malaria treatment. Additionally, a lack of baseline data or control group makes it difficult to assess the internal validity of these findings. However, the fact that this was a multi-year study strongly hypothesizes improved trends of CHW utilization similar to many studies conducted in the same geographical context corresponding to a non-reported significant change in the climatic conditions that are favourable for malaria transmission.

## Conclusions

The results of this study provide evidence that CHWs have the potential to be increasingly accepted and utilized by communities as part of a functional community health care delivery. In the context of malaria, this has the potential for improved management of uncomplicated fever-related malarial cases in rural Kenya, thereby reducing the burden on facility-based management through task-shifting. The chance of the malaria case being diagnosed by CHWs versus being diagnosed in a health facility was similarly high in periods 1–3 months following rainfall. This predictability presents opportunities for policy-targeted preparedness and control measures that would realize overall malaria case management. However, rainfall, tested under different regimes (current, lagged or cumulative) was not a predictor for diagnosing malaria by CHWs and in health facilities. Data on spatial hydrologic variability that is thought to influence local, village-scale mosquito abundance in areas of water-limited, seasonal malaria transmission was not available in this study. Therefore, it was only possible to hypothesize in village-level variability in the risk of malaria diagnosis. These findings will contribute to policy actions that may scale-up community health services in remote rural settings. Additionally, operational research is needed to understand the bidirectional knowledge, attitude and practices among CHWs and the communities they serve given the role that CHWs could play in improving access to malaria treatment (and other preventable childhood illnesses as well).

## Abbreviations

ACT: artemisinin-based combination therapy
AIDS: acquired immunodeficiency syndrome
CHW: community health worker
HIV: human immunodeficiency virus
MDG: millennium development goal(s)
OPD: Out-Patient Department
RDT: rapid diagnostic test
TRMM: tropical rainfall measurement mission
WHO: World Health Organization
